# Implementation of a Generative AI-Powered Digital Interactive Platform for Clinical Language Therapy in Children with Language Delay: A Pilot Study

**DOI:** 10.3390/life15101628

**Published:** 2025-10-18

**Authors:** Chia-Hui Chueh, Tzu-Hui Chiang, Po-Wei Pan, Ko-Long Lin, Yen-Sen Lu, Sheng-Hui Tuan, Chao-Ruei Lin, I-Ching Huang, Hsu-Sheng Cheng

**Affiliations:** 1 Department of Physical Medicine and Rehabilitation, Kaohsiung Medical University Hospital, Kaohsiung Medical University, Kaohsiung 807, Taiwan; joanna5421004@livemail.tw (C.-H.C.); chiang651@gmail.com (T.-H.C.); klin967@gmail.com (K.-L.L.); winereric383@gmail.com (Y.-S.L.); pj73010@hotmail.com (S.-H.T.); raykmuh03@gmail.com (C.-R.L.); 100311038@gms.tcu.edu.tw (I.-C.H.); 2 Medical Device Development Section, The Metal Industries Research & Development Centre, Kaohsiung 811, Taiwan; siquare@mail.mirdc.org.tw; 3 Department of Physical Medicine and Rehabilitation, School of Medicine, College of Medicine, Kaohsiung Medical University, Kaohsiung 807, Taiwan; 4 Department of Rehabilitation Medicine, Cishan Hospital, Ministry of Health and Welfare, Kaohsiung 842, Taiwan; 5 Department of Physical Medicine and Rehabilitation, Kaohsiung Municipal United Hospital, Kaohsiung 804, Taiwan

**Keywords:** language delay, autism spectrum disorder, early intervention, generative artificial intelligence, home-based rehabilitation

## Abstract

Early intervention is pivotal for optimizing neurodevelopmental outcomes in children with language delay, where increased language stimulation can optimize therapeutic outcomes. Extending speech–language therapy from clinical settings to the home is a promising strategy; however, practical barriers and a lack of scalable, customizable home-based models limit the implementation of this approach. The integration of AI-powered digital interactive tools could bridge this gap. This pilot feasibility study adopted a single-arm pre–post (before–after) design within a two-phase, mixed-methods framework to evaluate a generative AI-powered interactive platform supporting home-based language therapy in children with either idiopathic language delay or autism spectrum disorder (ASD)-related language impairment: two conditions known to involve heterogeneous developmental profiles. The participants received clinical language assessments and engaged in home-based training using AI-enhanced tablet software, and 2000 audio recordings were collected and analyzed to assess pre- and postintervention language abilities. A total of 22 children aged 2–12 years were recruited, with 19 completing both phases. Based on 6-week cumulative usage, participants were stratified with respect to hours of AI usage into Groups A (≤5 h, *n* = 5), B (5 < h ≤ 10, *n* = 5), C (10 < h ≤ 15, *n* = 4), and D (>15 h, *n* = 5). A threshold effect was observed: only Group D showed significant gains between baseline and postintervention, with total words (58→110, *p* = 0.043), characters (98→192, *p* = 0.043), type–token ratio (0.59→0.78, *p* = 0.043), nouns (34→56, *p* = 0.043), verbs (12→34, *p* = 0.043), and mean length of utterance (1.83→3.24, *p* = 0.043) all improving. No significant changes were found in Groups A to C. These findings indicate the positive impact of extended use on the development of language. Generative AI-powered digital interactive tools, when they are integrated into home-based language therapy programs, can significantly improve language outcomes in children who have language delay and ASD. This approach offers a scalable, cost-effective extension of clinical care to the home, demonstrating the potential to enhance therapy accessibility and long-term outcomes.

## 1. Introduction

Language development is a critical part of early childhood and serves as a foundational domain for cognitive, social, and academic growth [1,2]. Children with delayed language development often experience significant challenges, including language comprehension, expressive communication, and pragmatic use of language. Without appropriate intervention, these difficulties may persist into adulthood, with long-term impacts on education, employment, and psychosocial well-being [1,2].

Among children with language challenges, two major clinical groups are commonly encountered: those with idiopathic developmental language delay and those with autism spectrum disorder (ASD). While these populations differ in etiology and behavioral phenotype, they frequently exhibit overlapping impairments in language acquisition, particularly in expressive vocabulary, syntactic structure, and conversational pragmatics [1,2,3,4,5]. In ASD, language impairment is often accompanied by broader social–communicative deficits and restrictive–repetitive behaviors, whereas in idiopathic language delay, deficits are more circumscribed but can still interfere with learning and participation. Importantly, both groups benefit from early, intensive, and contextually rich language input [2,3,4,5].

Early identification and intervention during the critical developmental window that typically occurs before the age of six years are widely recognized as essential to optimizing neurodevelopmental outcomes [3]. In this period, the brain exhibits heightened neuroplasticity, which renders it particularly responsive to environmental input and therapeutic stimulation. Numerous studies show that early, intensive, and systematic language therapy can significantly improve language outcomes in children with developmental delays and ASD alike [2,3,4,5].

However, traditional center-based models of speech–language therapy can encounter difficulties. Children typically receive therapy once or twice per week, with each session lasting approximately 30 to 45 min. Their frequency and duration are constrained by therapist availability and institutional capacity. Furthermore, caregivers frequently encounter barriers, including limited time, lack of training, and insufficient guidance for implementing therapeutic techniques. These challenges can compromise the continuity and intensity of the language stimulation that is required to achieve optimal therapeutic outcomes.

To address these possible issues and facilitate caregiver work, expanding the scope of speech–language therapy to the home setting has emerged as a critical strategy. Home-based interventions enable frequent language stimulation and foster active caregiver involvement, enhancing the generalization of learned skills to everyday contexts. However, the clinical landscape lacks scalable, customizable, and user-friendly models of effective support for home-based language rehabilitation, particularly regarding adaptation to the unique needs of individual children and families.

Recent advancements in digital health and artificial intelligence (AI) provide promising opportunities for filling this gap. In particular, the integration of AI-powered digital interactive language therapy tools into home-based training platforms is a novel approach to supporting language development in children with delays [6]. Generative artificial intelligence (GenAI), which is able to personalize content, produce naturalistic dialogs, and adapt immediately to user performance, can prove particularly promising for tailoring language interventions to individuals. When deployed on tablets, AI tools can enable children to engage in meaningful, interactive rehabilitation activities at home, reinforcing and extending the effects of in-clinic therapy [7,8]. However, this use of GenAI raises important questions regarding safety, appropriateness, and interpretability considerations, which this study addresses through clinical validation.

This study aimed to evaluate a pediatric intervention that integrates three essential components: medical expertise from speech–language pathologists (SLPs), structured home-based therapeutic activities, and advanced digital technology. By bridging institutional care with at-home rehabilitation, this model was designed to strengthen language development and enhance therapeutic engagement.

The primary objective of this study was to assess the therapeutic efficacy of a generative AI-powered digital interactive platform designed to support home-based language therapy for children with language delay or autism spectrum disorder (ASD), through the comparison of pre- and post-intervention standardized language outcomes. The secondary objectives were to evaluate the usability, feasibility, and engagement patterns of the digital platform in real-world home settings; explore potential dose–response (threshold) relationships between cumulative usage duration and language improvement; and demonstrate how clinical speech–language expertise can be effectively integrated with AI-driven technology to establish a scalable framework for extending clinical care into the home environment [9,10,11,12,13].

## 2. Materials and Methods

### 2.1. Study Design and Participants

This was a two-phase, mixed-methods clinical investigation conducted in Kaohsiung Medical University Hospital and Kaohsiung Municipal United Hospital, tertiary-level medical institutions in Taiwan. This study evaluated the feasibility and therapeutic efficacy of a GenAI-powered interactive language therapy system for children with delayed language development, including children with ASD. The Institutional Review Boards (IRBs) of both institutions approved the study before its commencement (KMUHIRB-F(I)-20240036, KSVGH24-CT2-15; approval date: 11 March 2024). Recruitment and data collection were conducted from March 2024 through February 2025.

### 2.2. Diagnostic Classification

Diagnostic categories are reported using ICD-10-CM codes because, during the study period, both institutions’ electronic medical records, IRB documentation, and reimbursement systems operated under ICD-10 [14]. Moreover, the current Taiwan National Health Insurance (NHI) system is based on ICD-10, and therefore, all medical institutions nationwide adopt ICD-10 for clinical documentation, billing, and reporting. Clinical diagnoses were established by specialists based on standard criteria; ICD codes were applied for administrative classification. For transparency, we note that these ICD-10 categories correspond conceptually to the ICD-11 groupings of developmental speech or language disorders and autism spectrum disorder. The corresponding diagnostic categories based on ICD-10 codes are listed in Appendix A Table A1 [14,15].

All clinical diagnoses were established by board-certified specialists in physical medicine and rehabilitation, developmental pediatrics, or child psychiatry. Each diagnosis was based on standardized procedures that included structured behavioral observations, caregiver interviews, and age-appropriate developmental assessments, in accordance with current clinical practice guidelines.

Written informed consent was obtained from the participants’ legal guardians. In addition, verbal or nonverbal assent was secured from the children when developmentally appropriate. Eligible participants were children aged 2 to 12 years who had received a confirmed clinical diagnosis of language delay or autism spectrum disorder (ASD), corresponding to ICD-10 codes F80.1, F80.2, F80.4, F80.89, F84.0, F84.5, F84.8, or F84.9. Only children who demonstrated spontaneous verbal production and were actively participating in ongoing speech–language therapy were included. Participants were excluded if they lacked spontaneous speech, exhibited solely echolalic patterns without functional communication, or presented with severe articulation disorders that impeded intelligibility. Additional exclusion criteria included the inability to follow verbal instructions or the presence of severe emotional or behavioral disturbances that precluded effective engagement in the intervention.

### 2.3. Procedures

The study was conducted in two sequential phases designed to evaluate both the development and clinical implementation of the GenAI-powered interactive language therapy system. Phase I focused on the collection of standardized clinical language data to refine the AI-based system, while Phase II evaluated the usability and therapeutic effectiveness of the finalized software in home-based environments. Each phase was supervised by licensed speech–language pathologists (SLPs) and conducted in accordance with standardized clinical protocols to ensure the reliability and validity of the study outcomes.

• Phase I: Clinical Language Data Collection

All participants underwent standardized language assessments conducted by licensed SLPs. During routine speech–language therapy sessions, clinical language samples were recorded to form a corpus to optimize a GenAI-powered therapy system. A total of 2000 audio recordings were collected from all participants.

• Phase II: Home-Based Software Usability and Effectiveness Evaluation

After the initial data collection and software refinement processes, the selected participants were provided with tablets with the GenAI-enhanced interactive language therapy software to enable continuation of their clinical sessions at home. The caregivers were trained in the use of the software and received ongoing support. The home evaluation period for the device lasted a total of 6 weeks.

System logs automatically record each child’s engagement time. Total usage time represented the cumulative duration of active participation, excluding periods of inactivity, such as breaks, interruptions and application downtime. Based on cumulative usage across the 6-week intervention, participants were stratified into four usage groups: Group A: ≤5 h, Group B: 5 < h ≤ 10, Group C: 10 < h ≤ 15, and Group D: >15 h. A threshold of 15 h was chosen to represent sustained engagement. To evaluate longitudinal change, baseline (T0) was defined as the first 30 min of actively engaged use during week 1, and post-intervention (T1) as the final 30 min of engaged use during week 6. Inactivity was excluded by backend event logs.

The SLPs conducted weekly follow-up evaluations during therapy sessions. These included assessments of software usage, caregiver and participant feedback, and technical or usability concerns. Intervention effectiveness was evaluated by assessing language performance before and after using the home training program with clinical judgment and objective measures.

### 2.4. Language Therapy Software

The interactive language therapy tool used was a custom-designed iOS application for children with language delays and ASD. It was deployed on tablet devices as a home-based language rehabilitation program. The app includes four core modules: object imitation, cloze-picture tasks, storybook-based language modeling, and scenario-based dialog. Each module employs GenAI to enable real-time interaction. This study focused on object imitation and cloze-picture modules, which promoted language development through gamified therapy. All content was developed by licensed SLPs to ensure clinical appropriateness and therapeutic validity of the input.

### 2.5. Backend Architecture and GenAI Services

The system architecture integrated AI-powered cloud services for real-time, individualized interaction within the program, during which children received language prompts and responded verbally. These speech inputs and task-related metadata were transmitted to the backend server for real-time processing. The backend pipeline consisted of ASR (automatic speech recognition), a GenAI-based dialog system, and a TTS (text-to-speech) module, which enabled immediate therapeutic feedback and enhanced the system to adapt to the child’s language performance.

To enhance user engagement, the application featured vibrant animations and rich auditory feedback. Upon completing the task levels, the children received positive reinforcement animations, sounds, and virtual coins to exchange for gifts in the app to encourage task completion and sustain user interest. In addition, the application featured a backend system allowing parents to monitor their children’s usage patterns, including daily use and the number of completed levels. It also provided language development metrics, including the mean length of utterance (MLU), type–token ratio (TTR), single-word usage, and two-word combinations. To prevent excessive screen time, the application included a timer to enforce a 10 min break after every 30 min of continuous use.

The GenAI service was developed using Python (v3.10.13) and deployed on a FastAPI (v0.109.0) server framework, which offered high concurrency handling and low latency to meet the real-time demands of language-based interaction. Backend processes, including speech scoring and speech synthesis, were managed asynchronously using a message queue system, which ensured responsiveness and stability. The architecture had a modular design, providing scalability and flexibility for the seamless integration of various natural language processing (NLP) components, including speech recognition, semantic understanding, dialog generation, and speech output, providing a comprehensive pipeline for interactive language processing.

### 2.6. NLP and Language Processing Modules

For enhanced accuracy of the semantic interpretation of speech, the backend system implemented a hierarchical NLP engine comprising modules for part-of-speech tagging, intent classification, semantic parsing, and dialog state tracking. The system integrated traditional Chinese language processing tools, including Chinese Knowledge and Information Processing (CKIP) for word segmentation and syntactic analysis. A hybrid rule-based and intent-driven dialog state machine was created to guide user utterances toward contextually appropriate responses. Additionally, the NLP layer employed in-context learning strategies to dynamically select the most suitable large language model (LLM) in relation to the detected communicative intent and task context, enabling semantically relevant and context-aware response generation.

### 2.7. Speech Therapy Guidance Response

To effectively guide and support children’s speech during therapy, the system established a state machine framework mapping NLP component to the therapeutic strategies implemented through LLMs. In this state machine, a set of well-defined, commonly used guidance techniques of speech therapy is applied, including positive reinforcement, modeling, expansions, and extensions/expatiation. The system is continuously refined based on speech data collected through IRB-approved protocols, ensuring its clinical relevance and contextual appropriateness.

### 2.8. Interaction Logging and Data Capture

All user interactions were recorded and de-identified with a secure, anonymized participant identification system to ensure privacy protection. The backend system logs key indicators from each interaction session, including transcriptions, session duration, task completion rates, and specific items attempted. These data enable individual language performance and engagement to be tracked throughout the intervention, providing empirical evidence in support of therapeutic outcome evaluation and model refinement.

### 2.9. Language Output Evaluation

To systematically analyze children’s language development, the system incorporated an automated semantic and syntactic analysis framework based on standardized language assessment models. To classify lexical items into content words and function words, part-of-speech tagging was performed using CKIP. Language production was quantified using the total word count, morpheme count, and ratio of content to function words across lexical categories. Syntactic complexity and lexical diversity were measured using TTR, and MLU was used to indicate grammatical development. Both word-based (MLUw) and character-based (MLUc) versions of MLU were calculated. This analytic framework was a key basis for monitoring children’s language development and evaluating the intervention’s effectiveness.

### 2.10. Variables of Interest

This study examined both linguistic and engagement-related variables to evaluate the effectiveness and feasibility of the AI-assisted language therapy platform. The primary variables of interest were expressive language outcomes derived from pre- and post-intervention language samples, including total word count, total character count, type–token ratio (TTR), mean length of utterance (MLU, in words and characters), and the frequency of major lexical categories (nouns and verbs). These indicators collectively reflected participants’ lexical diversity, syntactic complexity, and expressive language growth.

The secondary variables of interest included user engagement and feasibility metrics, such as cumulative active usage time, session frequency, and caregiver-reported usability. These variables were analyzed to explore potential dose–response effects and to assess the practicality of sustained home-based intervention.

### 2.11. Assessment Tools

Language ability was evaluated at baseline and postintervention using standardized age-appropriate tools, as shown by the following:

Ages 2–3: Communication and Language Screening Test from Birth to Three Years Old for Chinese-Speaking Infant-Toddlers (CLST). The CLST was developed and standardized in Taiwan for preliminary linguistic assessment in Chinese-speaking infants and toddlers aged 0 to 3 years. Screening was conducted with caregiver interviews to identify children at high risk of language developmental delay. Normative data were established on the basis of a representative sample of 1236 infants and toddlers aged 0 to 3 years recruited from Northern, Central, Southern, and Eastern Taiwan and offshore islands. The test–retest reliability ranged from 0.91 to 0.99 across age groups, with an overall reliability of 0.99. Internal consistency ranged from 0.70 to 0.87 across age groups, with an overall value of 0.98, and inter-rater reliability was 0.99 [16].

Ages 3–6: Revised Preschool Language Scale—Chinese version. The Revised Language Disorder Assessment for Preschool Children is a standardized language assessment tool developed in Taiwan. It evaluates language ability in preschool-aged children between 3 and 6 years old. This tool was designed to assist professionals in screening and diagnosing language disorders in early childhood. While specific reliability and validity data have not been reported publicly, the assessment is widely utilized in clinical and educational settings across Taiwan. Its inclusion in multiple studies and practical applications supports its clinical utility and credibility [17].

Ages 6–12: Revised Language Assessment Battery for School-Aged Children. This is a standardized assessment tool developed in Taiwan, designed for children aged 6 to 12 years, that evaluates language ability. It supports professional screening and diagnosis of language disorders in school-aged populations. While no specific data on its reliability and validity are publicly available, the assessment is widely used in both clinical and educational settings in Taiwan. Its widespread integration into research and applied practice demonstrates its utility and credibility [18].

All of these tools are culturally adapted and validated for Mandarin-speaking populations, with reported reliability and construct validity in local pediatric cohorts. They comprehensively assess auditory comprehension, expressive language, vocabulary, articulation, and grammar [16,17,18].

### 2.12. Data Collection and Monitoring

All 2000 audio recordings were anonymized and securely stored for analysis. They were used to monitor changes in language output, vocabulary diversity, and syntactic complexity, at both T0 and T1 as defined above. Usage stratification into Groups A–D was also based on these backend logs. The data were reviewed by both automated analysis modules and licensed SLPs with over five years of clinical experience. Software usage data were also collected to assess engagement, frequency of use, and adherence to therapy. Data privacy and confidentiality were strictly maintained in accordance with institutional and national research ethics guidelines.

### 2.13. Statistical Analysis

All statistical analyses were performed using SPSS for Windows, version 29.0 (Released 2024; IBM Corp., Armonk, NY, USA). Given the pilot and feasibility design of this study and the limited group sizes, we employed nonparametric methods to provide assumption-light and interpretable results. Continuous variables are presented as means with standard deviations or medians with interquartile ranges (IQRs), and categorical variables as counts with percentages.

Between-group comparisons of baseline demographic and clinical characteristics were performed using the Kruskal–Wallis test for continuous variables (e.g., age, total usage time) and the chi-square test (or Fisher’s exact test when appropriate) for categorical variables (sex, diagnosis, hospital affiliation). Baseline language performance measures (T0) were also compared across the four usage groups using the Kruskal–Wallis test. Within-group comparisons of language outcomes between baseline (T0) and post-intervention (T1) were conducted using the Wilcoxon signed-rank test for paired nonparametric data. Exact two-sided *p*-values were calculated; because of the small sample size, discrete exact values (e.g., *p* = 0.043) may appear repeatedly across outcomes, reflecting the limited rank distributions.

A two-sided *p* < 0.05 was considered statistically significant. No formal sample size calculation was performed because of the feasibility design. Instead, analyses were intended to provide preliminary signals of potential efficacy to guide the design of future adequately powered randomized controlled trials.

## 3. Results

### 3.1. Overall Participant Characteristics

A total of 22 participants were initially enrolled; however, three discontinued therapy for personal reasons. The final analytic sample comprised 19 children who completed both phases of the investigation. After having stratified the 19 participants with respect to the total time spent using the GenAI-powered digital interactive language tool over the fixed 6-week period, the A–D groups resulted in five participants in group A, B and D, and four participants in group C. The cohort included children recruited from two tertiary hospitals: Kaohsiung Medical University Hospital (57.9%) and Kaohsiung Municipal United Hospital (42.1%). The mean age was 5.53 ± 1.81 years, and male participants predominated (68.4%). Approximately half of the participants had autism spectrum disorder (52.6%), while the remainder had developmental language delay (47.4%). The mean total usage time was 10.73 ± 8.59 h, reflecting substantial variability across the sample (Table 1).

### 3.2. Group-Wise Participant Characteristics

When stratified into Groups A–D according to cumulative usage time, the median age and sex distribution did not differ significantly among groups (*p* = 0.512 and *p* = 0.463, respectively). Similarly, the distribution of diagnostic categories (autism vs. language delay) was comparable (*p* = 0.368). However, there was a significant imbalance in hospital affiliation (*p* = 0.006). As expected, median usage hours increased progressively across the groups (A: 1.46 h; B: 6.96 h; C: 10.82 h; D: 16.53 h; *p* < 0.001) (Table 2).

### 3.3. Baseline (T0) Language Performance Across Groups

At baseline (T0), there were no statistically significant group differences in any of the measured language parameters, including total utterances, total words, total characters, lexical categories (nouns, verbs, adjectives, numerals, quantifiers, pronouns, adverbs, prepositions, conjunctions, auxiliary verbs, onomatopoeia), type–token ratio (TTR), mean length of utterance (MLU) in words and characters, and the longest five utterances (MLUw, MLUc). This indicates that the groups were relatively comparable in language performance prior to intervention (all *p* > 0.05) (Table 3).

### 3.4. Within-Group Comparisons of T0 and T1 Language Outcomes

These findings are summarized in Table 4. The evaluated linguistic outcomes included total word and character counts, type–token ratio (TTR) as an indicator of lexical diversity, the number of nouns and verbs produced, MLU in both words and characters, and the average words and characters across the five longest utterances.

Pre–post comparisons revealed distinct patterns across groups. In Groups A, B, and C, no statistically significant differences were observed between T0 and T1 across any of the measured outcomes (all *p* > 0.05). By contrast, participants in Group D, who engaged with the system for more than 15 h, demonstrated significant improvements across multiple language parameters, including total words, total characters, TTR, nouns, verbs, MLU, and both word- and character-based measures of the longest utterances (all *p* = 0.043). These results suggest a potential threshold effect, indicating that only children who engaged in extended and consistent use of the platform achieved meaningful gains in expressive language performance. The improvements were particularly notable in lexical diversity, reflected by TTR, and syntactic complexity, reflected by MLU. These findings indicate that extended and consistent use of the GenAI-enhanced therapy system (i.e., >15 h) is positively associated with marked improvements in expressive language output, particularly in lexical diversity and syntactic complexity [19,20]. The observed changes between T0 and T1 in Group D indicate the effectiveness of sustained home-based intervention using digital, AI-assisted tools for pediatric language rehabilitation.

## 4. Discussion

This pilot study investigated the implementation and effectiveness of a GenAI-powered digital interactive language therapy platform for use in children with delayed language development or ASD. The results provide preliminary evidence in support of the feasibility and therapeutic benefits of AI-assisted interventions in traditional speech–language therapy frameworks.

This study finds that children who used the AI-enhanced language therapy tool for more than 15 h had statistically significant improvements in multiple aspects of expressive language output, including total word and character counts, lexical diversity (as measured by the type–token ratio), frequency of noun and verb use, and utterance complexity (MLU and analysis of five longest utterances). These improvements were observed in a within-subject comparison of early (T0) and late (T1) usage sessions, strengthening the interpretation that the gains were attributable to the intervention.

These observations support prior findings of therapy intensity and frequency having a role in supporting language development in children with neurodevelopmental disorders [1,2]. Allowing children to perform language-rich tasks at home with the AI-powered system effectively increased the amount and continuity of their language stimulation. The integration of personalized feedback and naturalistic interaction with generative language modeling could have enhanced participant engagement and pragmatic skills, an area where traditional training is often limited or underemphasized, as pragmatic competence typically requires extensive conversational practice and interactive language activities [21].

The GenAI component in this digital therapy tool dynamically adapts prompts and content based on user response, simulating interactive dialog and offering semantically contingent feedback. This adaptability enables greater linguistic complexity, encouraging more diverse and grammatically structured output.

This study analyzed the actual usage patterns of the application among the participating children. We calculated usage coverage as a percentage, defined as (number of days the application was used/number of days the device was borrowed) × 100%, and found that 89% of children had usage coverage exceeding 30%. Backend data revealed that 58% of users increased their total number of spoken responses by more than 500. These findings indicate that the software engaged the children and facilitated increased opportunities for spoken language production.

The finding that only those with >15 h of use had significant gains underscores the importance of sustained engagement. Future software should incorporate strategies such as adaptive gamification, more enriched reward systems, and parent-mediated progress tracking to promote use.

The scalability and adaptability of this digital model are significantly advanced over conventional center-based services, often constrained by staffing, scheduling, and caregiver access limitations [10]. This study demonstrates that advanced AI technologies can be embedded into clinically informed software platforms and deployed on widely accessible hardware (e.g., tablets), offering a viable solution to bridge gaps in the delivery of pediatric rehabilitation services [9,10,22].

Our findings align with evidence that intensity and context-rich input underpin gains in early language intervention, including non-digital approaches. Parent-implemented programs and early-intervention models (e.g., Kruythoff-Broekman et al., 2019 [23]; Vermeij et al., 2023 [3]) emphasize everyday communicative contexts and caregiver mediation [3,23]. The present GenAI-assisted, home-based model complements these principles by scaling access, standardizing practice opportunities, and delivering semantically contingent, adaptive feedback, while preserving caregiver participation. Future work should directly compare digital and parent-mediated implementations and evaluate hybrid models.

Importantly, the broad age range of participants (2 to 12 years) introduces considerable developmental variability in cognitive, linguistic, and social capacities, which may influence responsiveness to digital intervention. Younger children may require more scaffolding and external modeling, while older children may be more capable of engaging independently with AI-guided tasks. Future studies should stratify outcomes by age group to better understand differential treatment effects. In addition, while the GenAI-enhanced system provides individualized and adaptive language input, it is not intended to replace social interaction, which remains essential for language development. Social communication inherently involves reciprocal exchanges, emotional attunement, and context-rich feedback—elements that are only partially replicable through digital systems. Accordingly, the platform should be viewed as a complement to rather than a substitute for caregiver- and peer-mediated language experiences. To improve the device’s validity and therapeutic impact, future software iterations should explore the inclusion of multi-user interaction modules, peer modeling scenarios, and parent–child co-use interfaces. These features could help simulate real-world communication environments and reinforce the social dimensions of language learning.

This study has several limitations that warrant consideration. First, the small and uneven group sizes limited the statistical power and generalizability of the findings and also restricted the feasibility of applying covariate-adjusted analyses such as ANCOVA. Although ANCOVA is generally recommended for controlling baseline differences, its assumptions cannot be reliably met when each group contains only 4–5 participants, rendering results unstable and potentially misleading. For this reason, we adopted assumption-light nonparametric methods, such as the Wilcoxon signed-rank test, which are more appropriate for small samples and provide clinically interpretable preliminary insights. As an exploratory pilot feasibility investigation, our primary aim was to identify preliminary signals of therapeutic benefit rather than provide definitive causal evidence. Larger and more demographically diverse cohorts will be necessary to validate and extend these results. Second, the absence of a randomized control group precludes definitive causal inferences regarding the observed improvements. Future studies should employ RCT designs to rigorously evaluate intervention efficacy. Third, variability in usage timing and duration driven by differences in caregiver facilitation may have introduced confounding effects. Standardized usage protocols, automated adherence tracking, and adaptive engagement strategies will be essential to improve implementation fidelity in future applications. Finally, we did not collect structured qualitative feedback from participants or caregivers, which limits insights into user experience and acceptability. Future trials should integrate mixed-method approaches, such as structured surveys and caregiver interviews, to refine both the therapeutic platform and its clinical implementation.

Building upon these preliminary findings, future research should expand the use of generative AI-based rehabilitation systems into multicenter trials to validate efficacy and generalizability across diverse linguistic and cultural populations. Longitudinal studies could further clarify the sustainability of language gains and the influence of continued engagement over time. Integrating multimodal AI features—such as prosody, facial expression, and gesture recognition—may also enhance ecological validity and promote pragmatic communication development. From a clinical perspective, the introduction of GenAI into pediatric language rehabilitation should be viewed as an assistive partnership rather than a replacement for speech–language pathologists (SLPs). The platform can help SLPs and caregivers extend structured therapy opportunities into the home setting, allowing children to continue practicing learned skills in an interactive and adaptive environment. Moreover, AI-assisted monitoring enables clinicians to remotely track each child’s engagement, verify adherence, and review automatically analyzed language variables—such as lexical diversity, utterance length, and syntactic complexity—to inform individualized treatment planning. This approach empowers both professionals and caregivers, fostering a more integrated continuum of care. Future implementation should also address ethical considerations, including data privacy, algorithmic transparency, and age-appropriate content design. Ultimately, this study provides an initial framework for clinically guided, ethically grounded integration of AI into language therapy—bridging professional expertise, caregiver involvement, and intelligent digital support to advance precision rehabilitation and equitable access to early intervention.

## 5. Conclusions

This pilot study provides preliminary results on the potential clinical utility of a GenAI-powered interactive platform for language development in children with language delay and ASD. Children who used the system for more than 15 h showed statistically significant improvements in vocabulary output, syntactic complexity, and utterance length. These findings are preliminary and subject to the limitations of the pilot design; nonetheless, they are encouraging and support the future validation of purpose-built digital platforms in rigorously controlled trials.

This system helps extend therapeutic intervention from the clinic to the home, offering a scalable, personalized, and cost-effective approach to pediatric speech–language rehabilitation. By enhancing continuity of care and empowering caregiver participation, such AI-assisted tools can broaden the reach of early intervention. These results suggest that integrating advanced AI technologies into rehabilitation frameworks has the potential to complement conventional therapy and lay a foundation for future large-scale clinical trials.

## Figures and Tables

**Table 1:** Participant characteristics.

Hospital, *n* (%)	Kaohsiung Medical University Hospital: 11 (57.9%)	Kaohsiung Municipal United Hospital: 8 (42.1%)
Sex, *n* (%)	Male: 13 (68.4%)	Female: 6 (31.6%)
Diagnosis, *n* (%)	Autism: 10 (52.6%)	Developmental Delay: 9 (47.4%)
Age, mean (±SD), years	5.53 ± 1.81	
Usage time, mean (±SD), h	10.73 ± 8.59	
Usage time groups	≤5 h (Group A):	5 participants
	5 < h ≤ 10 (Group B):	5 participants
	10 < h ≤ 15 (Group C):	4 participants
	>15 h (Group D):	5 participants

SD: standard deviation.

**Table 2:** Group-wise participant characteristics.

Group	A (≤5 h)	B (5–10 h)	C (10–15 h)	D (>15 h)	*p*-Value
*n*	5	5	4	5	
Age ^a^, median (IQR), years	6 (3.5)	5 (1.5)	5.5 (3.25)	6 (2.5)	0.512
Sex, *n*					
Male	2	4	3	4	
Female	3	1	1	1	0.463
Hospital, *n*					
KMUH	1	5	4	1	
United	4	0	0	4	0.006
Diagnosis, *n*					
Autism	3	1	3	3	
Delayed language development	2	4	1	2	0.368
Time ^a^, median (IQR), h	1.46 (2.01)	6.96 (2.61)	10.82 (3.74)	16.53 (14.77)	<0.001

KMUH: Kaohsiung Medical University Hospital; IQR: interquartile range; Age and time were analyzed using the Kruskal–Wallis test ^a^; categorical variables were analyzed using the chi-square test. The variable “time” indicates the actual cumulative active usage duration (in hours) objectively recorded by the system. It is presented descriptively to confirm group classification and participant engagement, not as an independent outcome variable.

**Table 3:** Group-wise comparison of T0 language data.

Group	A	B	C	D	*p*-Value
*n*	5	5	4	5	
Total utterances	35 (20)	38 (14)	24.5 (42)	35 (22)	0.700
Total words	83 (54)	79 (70)	84.5 (54)	58 (41)	0.537
Total characters	146 (69)	129 (146)	127 (92)	98 (69)	0.645
TTR	0.647 (0.1134)	0.776 (0.1983)	0.698 (0.1690)	0.594 (0.1050)	0.187
Nouns	39 (16)	43 (19)	27 (34)	34 (25)	0.529
Verbs	25 (13)	19 (40)	15.5 (4)	12 (22)	0.171
Adjectives	0 (0)	0 (0)	0 (0)	0 (2)	0.423
Numerals	0 (3)	2 (2)	0 (2)	1 (3)	0.609
Quantifiers	1 (4)	1 (2)	0.5 (1)	1 (2)	0.660
Pronouns	2 (7)	2 (4)	3 (5)	1 (3)	0.600
Adverbs	7 (4)	4 (4)	2.5 (4)	2 (4)	0.067
Prepositions	3 (3)	2 (5)	2.5 (3)	1 (1)	0.335
Conjunctions	3 (3)	0 (1)	0 (1)	1 (3)	0.324
Auxiliary verbs	0 (3)	0 (1)	0.5 (2)	1 (1)	0.635
Onomatopoeia	0 (3)	0 (1)	0 (0)	0 (1)	0.477
MLU (words)	2.80 (1.42)	1.73 (1.78)	2.27 (2.90)	1.83 (0.89)	0.565
MLU (characters)	4.60 (2.09)	3.15 (3.24)	3.52 (1.35)	2.94 (1.67)	0.466
Top 5 MLU (words)	6.40 (4.40)	4.60 (2.10)	4.90 (2.80)	3.60 (0.80)	0.246
Top 5 MLU (characters)	8.40 (4.60)	6.20 (3.00)	6.30 (2.75)	5.20 (1.50)	0.129

All values are presented as the median (interquartile range, IQR) unless otherwise specified. All variables were analyzed using the nonparametric Kruskal–Wallis test due to non-normal distribution. T0: The first 30 min of language data recorded in the initial software use; TTR: type–token ratio; MLU: mean length of utterance.

**Table 4:** Comparison of T0 (initial 30 min) and T1 (final 30 min) within each group.

Group	A		B		C		D	
*n*	5		5		4		5	
	T0	T1	T0	T1	T0	T1	T0	T1
Total utterances	35 (20)	45 (24)	38 (14)	45 (32)	24.5 (42)	43.5 (30)	35 (22)	46 (43)
*p*-value	0.343		0.686		0.068		0.176	
Total words	83 (54)	104 (42)	79 (70)	128 (69)	84.5 (54)	131.5 (78)	58 (41)	110 (61)
*p*-value	0.080		0.138		0.068		0.043 *	
Total characters	146 (69)	166 (75)	129 (146)	220 (115)	127 (92)	227.5 (137)	98 (69)	192 (112)
*p*-value	0.080		0.138		0.273		0.043 *	
TTR	0.6471 (0.1134)	0.7179 (0.1951)	0.7763 (0.1983)	0.6667 (0.2674)	0.6986 (0.1690)	0.7812 (0.2310)	0.5941 (0.1050)	0.7843 (0.2380)
*p*-value	0.138		0.893		0.465		0.043 *	
Nouns	39 (16)	54 (21)	43 (19)	48 (27)	27 (34)	70 (38)	34 (25)	56 (31)
*p*-value	0.138		0.416		0.068		0.043 *	
Verbs	25 (13)	40 (31)	19 (40)	39 (34)	15.5 (4)	33 (38)	12 (22)	34 (27)
*p*-value	0.225		0.225		0.144		0.043 *	
MLU (words)	2.80 (1.42)	3.47 (1.34)	1.73 (1.78)	3.33 (1.54)	2.27(2.90)	2.82 (0.89)	1.83 (0.89)	3.24 (0.96)
*p*-value	0.225		0.080		1.000		0.043 *	
MLU (characters)	4.60 (2.09)	5.33 (2.42)	3.15 (3.24)	6.25 (3.13)	3.52 (1.35)	4.92 (0.94)	2.94 (1.67)	5.54 (1.85)
*p*-value	0.345		0.080		0.066		0.043 *	
Top 5 MLU (words)	6.40 (4.40)	5.80 (5.40)	4.60 (2.10)	5.40 (1.20)	4.90 (2.80)	5.60 (1.55)	3.60 (0.80)	5.80 (2.50)
*p*-value	0.176		0.461		0.461		0.043 *	
Top 5 MLU (characters)	8.40 (4.60)	9.80 (6.80)	6.20 (3.00)	9.00 (2.30)	6.30 (2.75)	9.60 (2.65)	5.20 (1.50)	9.60 (4.80)
*p*-value	0.136		0.279		0.068		0.043 *	

All values are presented as the median (interquartile range, IQR) unless otherwise specified. All parameters were analyzed using the Wilcoxon signed-rank test for nonparametric paired data. * *p* < 0.05, statistically significant difference. T0: The first 30 min of language data recorded during initial software use; T1: The first 30 min of language data recorded during the last phase of software use; TTR: type–token ratio; MLU: mean length of utterance; IQR: interquartile range.

**Figure A1:**
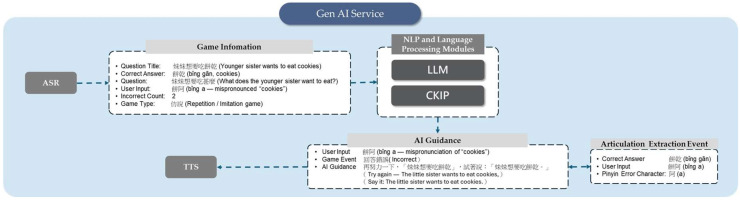
Generative AI service processing workflow.

**Figure A2:**
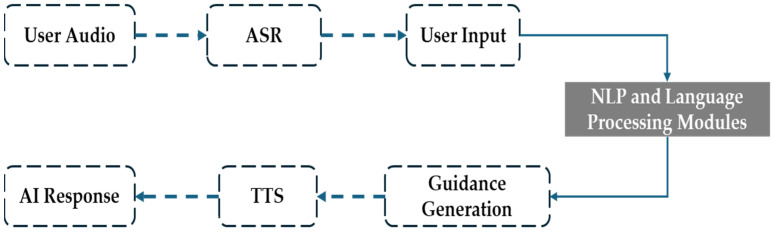
Generative AI service for dialog processing workflow.

**Figure A3:**
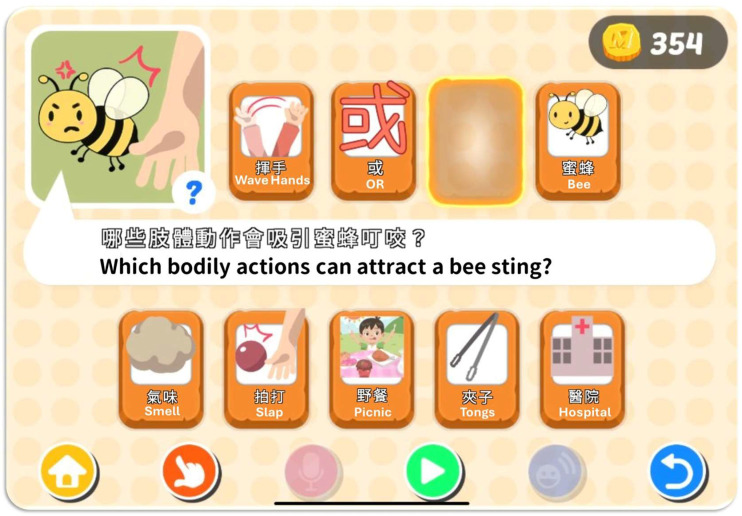
Representative interface of the cloze-picture tasks implemented in the language therapy software.

## Data Availability

The data presented in this study are available on request from the corresponding author due to privacy and ethical restrictions.

## Abbreviations

The following abbreviations are used in this manuscript:

ASD	Autism spectrum disorder
AI	Artificial intelligence
ASR	Automatic speech recognition
GenAI	Generative artificial intelligence
SLPs	Speech–language pathologists
ICD	International Classification of Diseases
MLU	Mean length of utterance
MLUw	Word-based mean length of utterance
MLUc	Character-based mean length of utterance
TTR	Type–token ratio
TTS	Text-to-speech
NLP	Natural language processing
CKIP	Chinese knowledge and information processing
LLM	Large language model
CLST	Chinese-speaking infant-toddlers
KMUH	Kaohsiung Medical University Hospital
IQR	Interquartile range
RCT	Randomized controlled trial

## Appendix A

**Table A1:** ICD-10 International Statistical Classification of Diseases and Related Health Problems 10th Revision.

ICD-10 Code	Full Diagnostic Name
F80.1	Expressive language disorder
F80.2	Receptive language disorder
F80.4	Speech and language development delay due to hearing loss
F80.89	Other developmental disorders of speech and language
F84.0	Childhood autism
F84.5	Asperger’s syndrome
F84.8	Other pervasive developmental disorders
F84.9	Pervasive developmental disorder, unspecified
